# Cellular Conductors: Glial Cells as Guideposts during Neural Circuit Development

**DOI:** 10.1371/journal.pbio.0060112

**Published:** 2008-04-29

**Authors:** Daniel A Colón-Ramos, Kang Shen

## Abstract

Glial cells rise to the forefront as key regulators that direct how neurons connect to one another when nervous system is built. A new article highlights the molecular and cellular mechanisms by which glia guide the exquisite architecture of the mammalian cerebellum.

The vertebrate nervous system is an astoundingly complex wiring network. Despite its complexity, the nervous system is also elegantly organized, and nowhere is this precise organization more evident than in the human brain. The human brain consists of an estimated 100 billion neurons interconnected through more than 100 trillion synapses, which are organized into the specific neural circuits that form the structural basis of information processing, storage, and ultimately behaviors.

One of the formidable questions in developmental neurobiology is how such a complex structure forms during development. How are the numerous cell types generated? How is the development of different cells coordinated spatially and temporally? What are the molecular and cellular “organizing principles” that help create such a precisely wired structure?

The answers to these questions will have a profound impact on both developmental biology and neuroscience. These questions will help us understand the events that coordinate the development of the most complex organ in the animal kingdom. Furthermore, our molecular knowledge of the development of the brain's architecture will help us understand the pathological implications of neurodevelopmental abnormalities. Animal models with specific developmental defects might also shed light on the contributions of particular brain structures to behavior.

## Building a Circuit

Neural circuit formation requires the intricate orchestration of multiple developmental events [1–3]. It begins with the specification of neuronal cell fate [4,5] followed by axon guidance. During axon guidance, a wide range of guidance cues act together to steer the growth cones toward their target field [6,7]. Once the target field is reached, however, axons still encounter many potential synaptic choices. The process whereby an axon discriminates between potential target choices and innervates the correct postsynaptic partner is known as synaptic specificity [8].

The question of how synapse specificity is directed is a formidable one in its own right. During synaptogenesis, proper synapse formation depends on pairing the right partners at the right density and at a specific subcellular location with respect to the dendrites. The assembly of presynaptic specializations also matches postsynaptic densities with respect to the identity of the neurotransmitter and the postsynaptic receptor type [2]. During development, this process occurs almost simultaneously in trillions of synapses, and the disruption of any of these neurodevelopmental steps affects synaptic communication and formation of functional neural circuits. While the circuitry of the vertebrate brain is subject to activity-dependent refinement, growing evidence suggests that the wiring events are genetically hard-wired at early stages of development [9–12]. We know remarkably little about the cellular and molecular mechanisms that coordinate this process of synaptic specificity.

## Synaptic Specificity in the Brain

On a cellular level, one might ask how pre- and postsynaptic cells reliably meet each other and choose each other as partners. At least two different scenarios have been proposed: the “dating” scenario and the “arranged marriage” scenario. In the “dating” mode, mutual attraction between the pre- and postsynaptic cells leads to the specific association between synaptic partners. In the “arranged marriage mode” however, a third cell can function as a guidepost to coordinate the innervation. This guidepost cell attracts both pre- and postsynaptic partners, enabling them to choose each other [13].

Experimental evidence from two studies in the nematode Caenorhabditis elegans supports the synaptic guidepost hypothesis. The worm egg-laying motor neuron HSNL forms synapses with its postsynaptic target muscles. The recognition between HSNL and its targets is mediated by the adjacent guidepost epithelial cells [14,15]. In the C. elegans nerve ring, two interneurons, RIA and AIY, reliably innervate each other at stereotyped locations. It turns out that a pair of nearby glia cells serve as guideposts for the innervation of these two interneurons [16].

Two examples of synaptic guideposts have also been reported in vertebrate systems. The transient population of Cajal-Retzius cells in the hippocampus serves as a placeholder to facilitate the meeting of the appropriate pre- and postsynaptic cells [17]. Also, during the development of the mammalian cortex, the subplate neurons display a similar guidepost function to arrange the marriage between the thalamic axons and the cortical neurons of layer 4 [18]. The significance of these guidepost cells was demonstrated by ablating the guidepost cells and showing a synaptic connectivity defect in ablated animals [17,19]. The cellular basis of how the guidepost cells associate with both synaptic partners was not explored in these studies because of the daunting complexity of the hippocampus and cortex. However, certain areas of the brain, such as the cerebellum, have neatly organized circuits that form uniform and stereotyped patterns. This cytoarchitecture facilitates in vivo studies aimed at understanding how this wiring precision is achieved [20].

The cerebellum is the area of the brain that integrates sensory perception and motor control. Precise neural connectivity is required for the cerebellum to link the sensory inputs (from the spinocerebellar tract) with the motor responses (from the motor cortex), and incorrect integration of these pathways results in impaired movement and motor coordination.

The wiring precision of the cerebellum is evident at two levels. First, neurons with different identities accurately select their synaptic partners from an array of potential choices. In making these synaptic choices, cellular contact with other neurons is not sufficient for synapse formation, and synaptic connections form at discrete subcellular regions of axons and dendrites. Second, synapses between particular neurons form at stereotyped locations. This selection of stereotyped locations gives rise to a highly organized, three-dimensional array of synaptic networks in the cerebellum [20].

**Figure 1:**
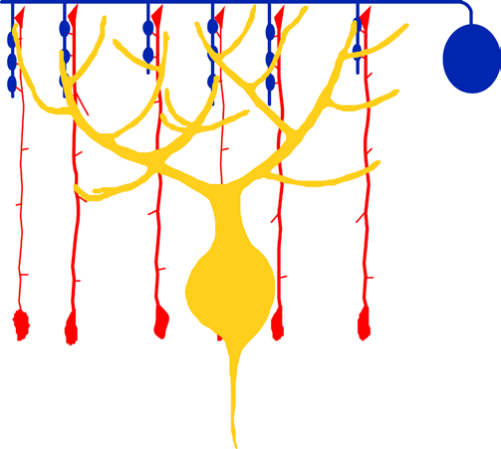
The Bergmann Glia Fibers Orchestrate the Precise Innervation of Stellate Axons to the Purkinje Dendrites Purkinje neurons (yellow) receive GABAergic inputs from stellate interneurons (blue) exclusively at the dendrites. This precision at the level of partner selection and subcellular localization of synapses is critical for the proper functioning of these cerebellar GABAergic circuits. How is this precision directed during development? In this issue of *PLoS Biology*, Ango et al. report that Bergmann glia (red) are the central orchestrators in the assembly of this circuit. Bergmann glia act as guideposts, directing the stellate interneuron process to their Purkinje neuron targets and coordinating the development of this precisely wired circuit.

For instance, Purkinje neurons, which are the sole output neurons from the cerebellar cortex, receive GABAergic inputs from two cell types: basket and stellate interneurons (Figure 1). This innervation reflects specificity at the level of partner selection. Furthermore, basket and stellate interneurons not only innervate Purkinje interneurons specifically, but they do it with subcellular precision: basket cells innervate Purkinje cells at their somata axon initial segments, whereas stellate cells do so at Purkinje cell dendrites [20] (Figure 1). This innervation reflects specificity at the level of stereotyped location. Although the specificity of these neural connections is well documented, the cellular and molecular mechanisms that underlie the development of such organized synaptic structures remained unknown until recently.

Work from Josh Huang's lab, presented in this issue of PLoS Biology [21] provides mechanistic insights on how this specificity is achieved—and the answers have been surprising. Standing in the spotlight as the orchestrator of the development of these important circuits is a member of one of the most overlooked cells in the nervous system: a glial cell.

## Glia and Neurodevelopment

The nervous system consists of two major cell types: neurons and glia. Although glia constitute 90% of cells in the human brain, they seldom share the limelight with their neuronal cousins. The word glia is Greek for “glue,” and these cells are generally thought of as a tissue scaffold, passively supporting the business-end of the nervous system–neurons [22].

Nonetheless, glia are far from passive scaffolds as they actually play critical roles in the development and function of the nervous system. Glia provide trophic support that is essential for neuron survival and homeostasis, and they regulate the production of neurons by modulating neuronal precursor divisions. They monitor neurotransmitter accumulation at the synaptic cleft and contribute to neuronal homeostasis through the release of growth and metabolic factors. Finally, they direct neuronal connections by directing axon pathfinding, promoting synaptogenesis, modulating dendrite morphology, and pruning excess axons [23].

Most of this knowledge on the role of neuro–glia interactions has come from studies conducted in the peripheral nervous system of vertebrates, from in vitro systems using dissociated neuronal cultures and from invertebrate model organisms [23]. It has been much harder to assess the in vivo function of glia in the central nervous system (CNS) of vertebrates. Do glia play a role in orchestrating the innervation of the brain?

Work from Ango et al. [21] indicates that they do. One prominent type of glial cells in the cerebellum, called Bergmann glia (BG), forms an ornate and highly organized meshwork of radial processes in the cerebellar cortex. This striking architecture has long been recognized and even hypothesized to play a role in the development of cerebellar neural circuits [24]. However, the role of the BG in directing the stereotyped development of the cerebellar circuits was not experimentally demonstrated.

Using green fluorescent protein bacterial artificial chromosome (GFP BAC) transgenic reporter mice, Ango et al. were able to determine the role of BG in directing the innervation of stellate and Purkinje cells. Stellate cells innervate the Purkinje neurons exclusively in the dendrite, and this precision at the level of partner selection and subcellular localization of synapses is critical for the proper functioning of these cerebellar GABAergic circuits. Ango et al. observed that stellate cells associated with BG during development, and followed the glia process by extending their axon through the curving contours of the BG fibers. By following the guidepost BG fibers, stellate cell processes are able to reach their targets: the dendrite of the Purkinje neurons (Figure 1).

Ango et al. also found the factor required in both BG and stellate cells for the proper development of this circuit—the L1 family immunoglobulin cell adhesion molecule, CHL1. Interestingly, previous work from the Huang lab had shown that another member of the L1 family, neurofascin186, is required for the specification of another part of this GABAergic circuit: the innervation of the Basket cells to the Purkinje cell at the axon initial segment [25]. This molecular characterization of the cerebellar GABAergic circuit suggests that different members of the L1CAM protein family contribute to circuit formation through their cell-specific expression in subsets of neurons and glia.

## Significance and Future Directions

In the brain, multiple developmental events are simultaneously orchestrated resulting in the innervation of pre- and postsynaptic partners at discrete neural coordinates. Cell–cell recognition events might account for the specificity at the level of partner selection, but how is this specificity directed with subcellular precision? Why is it that contact between potential partners is not sufficient for synaptic formation in one subcellular region, but it is in another? How are these “meeting points” between potential synaptic partners organized in the complex three-dimensional lattice of the human brain?

We do not have the complete answers to these questions; however, the work from Ango et al., together with that of other colleagues in this field, is starting to provide a conceptual framework for understanding how these processes could be orchestrated in vivo. Astrocytes have long been shown to have an intimate relationship with synapses. For instance, astrocytes have been shown to secrete factors that direct synaptogenesis in vitro and in vivo in both vertebrates and invertebrates [16,26].

The studies by Ango et al. provide novel insight into how astrocytes can orchestrate the precise development of stereotyped circuits in the mammalian brain. Given the close anatomical and functional relationships between glia and neurons, it is possible that the findings of Ango et al. could be a mechanism that's generalizable to other neural circuits, whereby glia act as key regulators by directing pre- and postsynaptic target interaction and the innervation of circuits in complex cellular environments.

## Abbreviations

BG: Bergmann glia
